# Gatifloxacin versus ceftriaxone for uncomplicated enteric fever in Nepal: an open-label, two-centre, randomised controlled trial

**DOI:** 10.1016/S1473-3099(15)00530-7

**Published:** 2016-05

**Authors:** Amit Arjyal, Buddha Basnyat, Ho Thi Nhan, Samir Koirala, Abhishek Giri, Niva Joshi, Mila Shakya, Kamal Raj Pathak, Saruna Pathak Mahat, Shanti Pradhan Prajapati, Nabin Adhikari, Rajkumar Thapa, Laura Merson, Damodar Gajurel, Kamal Lamsal, Dinesh Lamsal, Bharat Kumar Yadav, Ganesh Shah, Poojan Shrestha, Sabina Dongol, Abhilasha Karkey, Corinne N Thompson, Nga Tran Vu Thieu, Duy Pham Thanh, Stephen Baker, Guy E Thwaites, Marcel Wolbers, Christiane Dolecek

**Affiliations:** a Oxford University Clinical Research Unit, Patan Academy of Health Sciences, Kathmandu, Nepal; b Global Antibiotic Resistance Partnership, Kathmandu, Nepal; c The Hospital for Tropical Diseases, Wellcome Trust Major Overseas Programme, Oxford University Clinical Research Unit, Ho Chi Minh City, Vietnam; d Patan Hospital, Patan Academy of Health Sciences, Kathmandu, Nepal; e Centre for Tropical Medicine and Global Health, Nuffield Department of Medicine, Oxford University, Oxford, UK; f Department of Zoology, Oxford University, Oxford, UK; g Civil Services Hospital, Kathmandu, Nepal; h the London School of Hygiene & Tropical Medicine, London, UK

## Abstract

**Background:**

Because treatment with third-generation cephalosporins is associated with slow clinical improvement and high relapse burden for enteric fever, whereas the fluoroquinolone gatifloxacin is associated with rapid fever clearance and low relapse burden, we postulated that gatifloxacin would be superior to the cephalosporin ceftriaxone in treating enteric fever.

**Methods:**

We did an open-label, randomised, controlled, superiority trial at two hospitals in the Kathmandu valley, Nepal. Eligible participants were children (aged 2–13 years) and adult (aged 14–45 years) with criteria for suspected enteric fever (body temperature ≥38·0°C for ≥4 days without a focus of infection). We randomly assigned eligible patients (1:1) without stratification to 7 days of either oral gatifloxacin (10 mg/kg per day) or intravenous ceftriaxone (60 mg/kg up to 2 g per day for patients aged 2–13 years, or 2 g per day for patients aged ≥14 years). The randomisation list was computer-generated using blocks of four and six. The primary outcome was a composite of treatment failure, defined as the occurrence of at least one of the following: fever clearance time of more than 7 days after treatment initiation; the need for rescue treatment on day 8; microbiological failure (ie, blood cultures positive for *Salmonella enterica* serotype Typhi, or Paratyphi A, B, or C) on day 8; or relapse or disease-related complications within 28 days of treatment initiation. We did the analyses in the modified intention-to-treat population, and subpopulations with either confirmed blood-culture positivity, or blood-culture negativity. The trial was powered to detect an increase of 20% in the risk of failure. This trial was registered at ClinicalTrials.gov, number NCT01421693, and is now closed.

**Findings:**

Between Sept 18, 2011, and July 14, 2014, we screened 725 patients for eligibility. On July 14, 2014, the trial was stopped early by the data safety and monitoring board because *S* Typhi strains with high-level resistance to ciprofloxacin and gatifloxacin had emerged. At this point, 239 were in the modified intention-to-treat population (120 assigned to gatifloxacin, 119 to ceftriaxone). 18 (15%) patients who received gatifloxacin had treatment failure, compared with 19 (16%) who received ceftriaxone (hazard ratio [HR] 1·04 [95% CI 0·55–1·98]; p=0·91). In the culture-confirmed population, 16 (26%) of 62 patients who received gatifloxacin failed treatment, compared with four (7%) of 54 who received ceftriaxone (HR 0·24 [95% CI 0·08–0·73]; p=0·01). Treatment failure was associated with the emergence of *S* Typhi exhibiting resistance against fluoroquinolones, requiring the trial to be stopped. By contrast, in patients with a negative blood culture, only two (3%) of 58 who received gatifloxacin failed treatment versus 15 (23%) of 65 who received ceftriaxone (HR 7·50 [95% CI 1·71–32·80]; p=0·01). A similar number of non-serious adverse events occurred in each treatment group, and no serious events were reported.

**Interpretation:**

Our results suggest that fluoroquinolones should no longer be used for treatment of enteric fever in Nepal. Additionally, under our study conditions, ceftriaxone was suboptimum in a high proportion of patients with culture-negative enteric fever. Since antimicrobials, specifically fluoroquinolones, are one of the only routinely used control measures for enteric fever, the assessment of novel diagnostics, new treatment options, and use of existing vaccines and development of next-generation vaccines are now a high priority.

**Funding:**

Wellcome Trust and Li Ka Shing Foundation.

## Introduction

Enteric (typhoid) fever, a systemic infection caused by the *Salmonella enterica* serovars Typhi and Paratyphi A, B, and C, is a leading cause of febrile disease in many low-income countries. 27 million new infections and more than 200 000 deaths are estimated to be attributable to enteric fever worldwide each year.^1^, ^2^ In Kathmandu, the capital of Nepal and the setting of this study, the burden of enteric fever is particularly high, and is the leading cause of febrile bacterial disease in adults and children.^3^, ^4^

**Research in context**

**Evidence before this study**

We searched MEDLINE, PubMed, and Scopus without date restrictions for English-language articles with the search terms “randomized controlled trial” (“RCT” or “randomized* control* trial*”) AND “typhoid fever”, “enteric fever”, AND “ceftriaxone”. We also noted relevant articles outlined in a Cochrane review, and a meta-analysis, of fluoroquinolones versus other antimicrobials in the treatment of enteric fever. We identified 11 randomised trials that used intravenous ceftriaxone in one of their treatment groups. The selected trials had small sample sizes (ranging from 15 to 43 patients) and the definitions of outcomes for the primary and secondary outcomes were not standardised. Three trials gave ceftriaxone for 7 days in one of their groups (for 73 patients), the same duration as in our trial, but the drug dose was variable. The mean fever clearance times ranged from 3·9 days to 5·4 days, the number of clinical failures ranged from none to six, and the number of relapses ranged from one to four in these small trials.

**Added value of this study**

Our data augment previous findings, predicting that ceftriaxone is safe and effective for the treatment of enteric fever and out-performs gatifloxacin, with only 7% of culture-positive patients failing treatment, and a median fever clearance time of 2·78 days. However, our study, by contrast with the outlined studies, also investigated the clinical outcome in culture-negative patients with suspected enteric fever—in this group, gatifloxacin out-performed ceftriaxone with median fever clearance times of 1·12 days and 3·03, respectively. Furthermore, our work is the first to describe the clinical implications of fluoroquinolone-resistant *Salmonella enterica* serovar Typhi.

**Implications of all the available evidence**

In view of the emergence of fluoroquinolone-resistant *S* Typhi in this setting and the poor efficacy of ceftriaxone in the culture-negative group, we advocate better diagnostic testing for febrile diseases in low-income countries, and suggest that fluoroquinolones are no longer effective for treatment of enteric fever in Nepal.

Resistance and reduced susceptibility to antimicrobials are the major challenges to successful treatment of enteric fever.^5^, ^6^ We have previously reported^3^, ^7^ a high prevalence of *S* Typhi and *S* Paratyphi A strains in Nepal that show resistance against the quinolone nalidixic acid (minimum inhibitory concentration [MIC] ≥256 μg/mL) with a corresponding decreased susceptibility against fluoroquinolones such as ciprofloxacin (MIC ≥0·125 μg/mL).^3^, ^7^ Ceftriaxone, a parenteral, third-generation cephalosporin, is a common empirical therapy for febrile disease in endemic enteric fever locations, and is used for the treatment of enteric fever in south Asia and other regions where nalidixic acid-resistant strains predominate. Furthermore, ceftriaxone is also advocated for the treatment of travellers returning with enteric fever from areas of enteric fever endemicity.^8^, ^9^ Investigators for three randomised controlled trials have compared fluoroquinolones with ceftriaxone for treatment of enteric fever.^10^, ^11^, ^12^ Their findings generally favoured the fluoroquinolones, but the studies were insufficiently powered (only 15 to 25 patients per treatment group) to reach significance and data for the prevalence of nalidixic acid-resistant strains were not reported.^13^

Intravenous therapy is expensive and difficult to give reliably (particularly to outpatients) in most countries where enteric fever is endemic; therefore, effective oral antimicrobials are more practical for treatment of this disease. Previously, we have shown that even without reported resistance, the oral third-generation cephalosporin, cefixime, did poorly in Nepalese patients with enteric fever—treatment failure was reported in 26 (37%) of 70 patients receiving cefixime versus three (3%) of 88 patients receiving gatifloxacin.^14^ Conversely, we have also shown in Nepalese and Vietnamese children and adults with uncomplicated enteric fever that the fourth-generation 8-methoxy-fluoroquinolone gatifloxacin is safe and effective despite an increase in prevalence of *S* Typhi strains with reduced ciprofloxacin susceptibility.^14^, ^15^, ^16^, ^17^

Therefore, because third-generation cephalosporins are generally associated with slow clinical improvement and high relapse burden,^18^, ^19^ and 7 days of oral gatifloxacin is associated with rapid fever clearance (≤4 days) and low relapse burden, we postulated that gatifloxacin is superior to ceftriaxone in treating enteric fever, and did a study to test this hypothesis.

## Methods

### Study design and participants

We did an open-label, randomised, controlled, superiority trial at Patan Hospital and the Civil Services Hospital in the Kathmandu valley, Nepal. The study protocol was reviewed and approved by the Ethics Committee of the Nepal Health Research Council and the Oxford Tropical Research Ethics Committee (UK).

We screened children aged 2–13 years and adults aged 14–45 years with suspected enteric fever. The criteria for suspected enteric fever were body temperature at least 38·0°C for 4 days or more without a focus of infection, as assessed by physical examination and laboratory tests, and as previously described.^14^, ^16^, ^17^ Patients were excluded if they were pregnant; had diabetes mellitus, signs of severe infection (eg, obtundation, shock, clinical jaundice, or active gastrointestinal bleeding), or a history of hypersensitivity to either of the trial drugs; or had been given a fluoroquinolone, a third-generation cephalosporin, or a macrolide within the previous week. Patients who had received chloramphenicol, amoxicillin, or co-trimoxazole could be included, provided the treating clinician reported no clinical response. Written, informed consent to participate in the study was required from all patients. For patients younger than 18 years, we obtained written, informed consent from their parent or an adult guardian.

### Randomisation and masking

We randomly assigned patients (1:1) without stratification to 7 days of treatment with either oral gatifloxacin (10 mg/kg) once per day or intravenous ceftriaxone (60 mg/kg up to a maximum of 2 g for patients aged 2–13 years or 2 g for patients aged ≥14 years) once per day. The randomisation list was computer-generated with blocks of four and six (with equal probability) and maintained by a clinical trials pharmacist. We concealed treatment allocations inside opaque sealed envelopes, which were numbered sequentially to correspond to patient enrolment numbers. Envelopes were kept in a locked drawer and were opened in strictly numerical order by a study clinician (who had previously screened the patients and obtained consent). Treatment allocation was open-label; masking was not possible because of a difference in the administration route of the two drugs.

### Procedures

Gatifloxacin 400 mg tablets (Square Pharmaceuticals, Bangladesh) were weighed and cut at a dose of 10 mg/kg once per day. Ceftriaxone (Powercef, 1000 mg injection vial, Wockhardt Ltd, India), was injected slowly over 10 min once per day. Patients received the first dose (on day 1) of the study drug in hospital to monitor for anaphylaxis. Patients receiving ceftriaxone were discharged with an intravenous cannula in situ and had a new cannula inserted on day 4 of treatment. Home treatment was monitored by trained community medical auxiliaries (CMAs), as described in previous studies.^14^, ^16^, ^17^ A CMA visited each patient assigned to treatment twice per day for at least 10 days or until the patient was asymptomatic. The CMAs gave the drugs, and recorded drug doses, administration times, oral temperatures, symptoms, and potential adverse effects in a standard case-record form.

We measured complete blood count, serum creatinine, liver-function parameters (total bilirubin, aspartate aminotransferase, and alanine aminotransferase), and serum glucose at enrolment and on day 8 of treatment. We did a finger-prick test for glucose each day on days 2–7 after randomisation, and measured random serum glucose on day 8, day 15, and at 1 month.

We took blood from all patients (3 mL from those aged <14 years; 8 mL from those aged ≥14 years) for bacterial culture at enrolment and on day 8 after randomisation if *S* Typhi or *S* Paratyphi were isolated at enrolment, or if their symptoms suggested a clinical relapse. We inoculated blood samples from adults into media-containing tryptone soya broth and sodium polyanethol sulphonate, up to a total volume of 50 mL. We used BactecPeds Plus culture bottles (Becton Dickinson, New Jersey, USA) for paediatric blood samples. Culture results were reported for up to 7 days; positive bottles were subcultured onto blood, chocolate, and MacConkey agar, and colonies presumptive of salmonella were identified using standard biochemical tests and serotype-specific antisera (Murex Biotech, Dartford, UK). We measured antimicrobial sensitivities by the modified Kirby-Bauer disc diffusion method with zone size interpretation based on guidelines from the Clinical and Laboratory Standards Institute.^20^ Antimicrobial discs tested were ceftriaxone (30 μg), ciprofloxacin (5 μg), gatifloxacin (5 μg), and nalidixic acid (30 μg). MICs against these antimicrobials were measured by Etest, according to the manufacturer's recommendations (BioMérieux, France). All patients were requested to attend a clinic at Patan Hospital on day 8, day 15, and 1 month, 3 months, and 6 months after randomisation for clinical assessments and stool culture.

### Outcomes

The primary endpoint of this trial was a composite of treatment failure, defined as the occurrence of at least one of the following events: fever clearance time (ie, time from the first dose of a study drug until the temperature fell to ≤37·5°C and remained there for at least 2 days) more than 7 days after treatment initiation; the need for rescue treatment as judged by the treating physician (the recommended rescue treatment was azithromycin; however, any treatment other than the assigned treatment was acceptable); blood-culture positivity for *S* Typhi or *S* Paratyphi on day 8 of treatment (microbiological failure); culture-confirmed or syndromic enteric fever relapse within 28 days of treatment initiation; or development of any enteric fever-related complication (eg, clinically significant bleeding, a fall in the patient's Glasgow Coma Score, perforation of the gastrointestinal tract, or admission to hospital) within 28 days after treatment initiation. Time to treatment failure was defined as the time from the first dose of treatment until the date of the earliest failure event; patients without a failure event were censored on day 28 or the date of their last follow-up.

Secondary endpoints were fever clearance time only; time to relapse until day 28 or at any time during follow-up; confirmed and syndromic relapse until day 28; confirmed or syndromic relapse at any time; and faecal carriage of *S* Typhi or *S* Paratyphi at 1 month, 3 months, or 6 months after randomisation assessed in culture-positive patients only. We calculated fever clearance times electronically using temperatures recorded twice per day. We treated these times as interval-censored outcomes to show that fever clearance was known to have occurred at some unknown point in the interval from the last febrile temperature assessment until the first afebrile assessment. We treated patients without fever clearance or relapse as right-censored.

Safety and adverse events were assessed each day by the CMAs at the patient's home, by giving a symptom questionnaire and simple physical examinaton. Any patient who had unexpected symptoms was assessed by a study clinician in the hospital. Each patient was also seen by the study clinician in the hospital on the scheduled follow-up days and asked about any symptoms, and a physical examination was undertaken to assess for possible adverse events.

### Statistical analysis

In this study, we aimed to address the hypothesis that gatifloxacin was superior to ceftriaxone. On the basis of our previous data,^17^ we predicted that about 7% of patients with a positive culture given gatifloxacin would have treatment failure. To detect an increase in the risk of failure by 20% (from 7% to 27%) in the ceftriaxone group with 80% power at the two-sided 5% significance level, and allowing for a 10% loss to follow-up, we calculated that a sample size of 120 culture-positive patients (60 per treatment group) was needed. We assumed a culture-positive rate of at least 40%, and designed the trial to randomly assign 300 patients to treatment.

For treatment failure, we based the comparison of the absolute risk of treatment failure until day 28 on Kaplan-Meier estimates and corresponding standard errors according to Greenwood's formula.^21^ We used survival methods for the analysis of the time to treatment failure, fever clearance time, and time to relapse. For the times to treatment failure and relapse, we used the Kaplan-Meier method to calculate the cumulative incidence of events and Cox regression models with treatment group as the only covariate used for comparison between treatment groups. For the interval-censored fever clearance times, we used the non-parametric maximum likelihood estimator (NPMLE) to estimate their distribution, and parametric Weibull accelerated failure time models for the estimation of quantiles of the fever clearance time in each group and for the comparison between groups.^21^ We based median (IQR) calculations of fever clearance times on models for each treatment group separately, and acceleration factors on models that included treatment as the only covariate.

We undertook all analyses for each of the three main analysis populations: a modified intention-to-treat (ITT) population (consisting of all randomised patients who received at least one dose of study treatment and did not have a confirmed alternative diagnosis), and the subpopulations with either confirmed blood-culture positivity, or blood-culture negativity. Treatment failure and fever clearance time were also assessed in predefined subgroups (age <16 years; age ≥16 years; female; male; recruited before or after April, 2013; MIC against ciprofloxacin <0·12 μg/mL, 0·12–2·00 μg/mL, or >2·00 μg/mL; MIC against gatifloxacin ≤1·00 μg/mL or >1·00 μg/mL; *S* Typhi infection; and *S* Paratyphi infection). We tested for heterogeneity of treatment effects between subgroups with a Cox regression model (for analysis of treatment failure) or a Weibull accelerated failure time model (for fever clearance times) that included an interaction term between the treatment and the subgrouping variable. We did all analyses using the statistical software R version 3.0.1,^22^ based on available data without imputation of missing data. The safety of the trial was overseen by an independent data and safety monitoring board. This trial was registered at ClinicalTrials.gov, number NCT01421693.

### Role of the funding source

The funder of the study had no role in study design, data collection, data analysis, data interpretation, or writing of the report. The corresponding author had full access to all the data in the study and had final responsibility for the decision to submit for publication.

## Results

**Figure 1:**
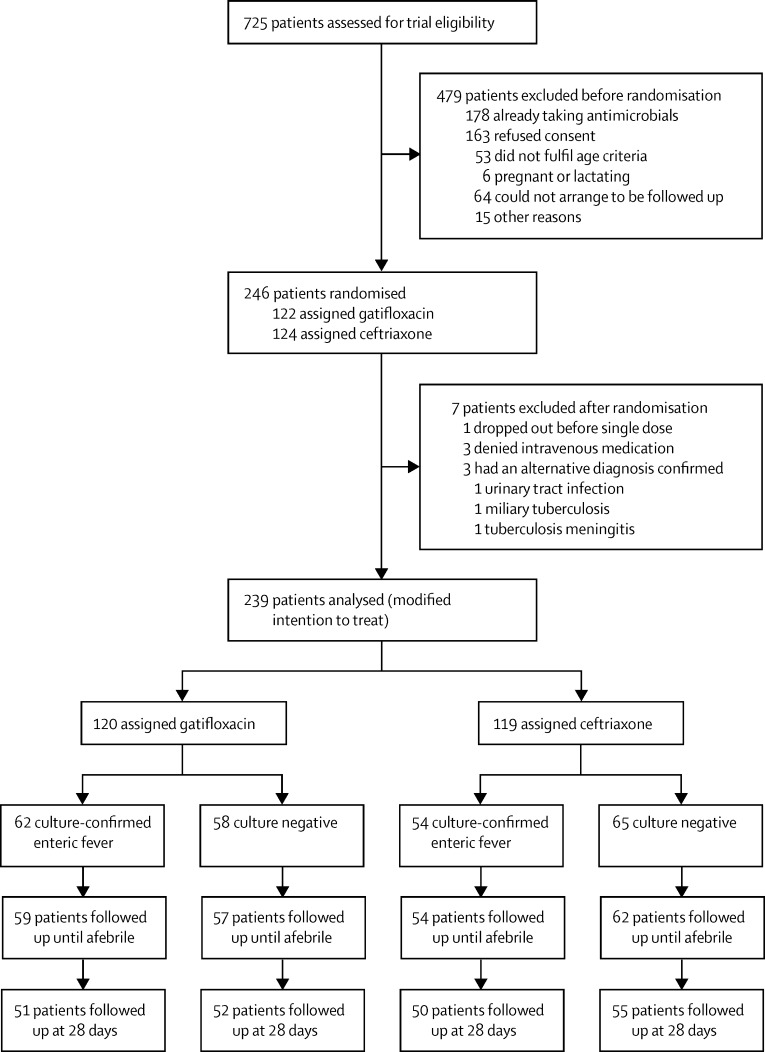
Trial profile *Salmonella enterica* Typhi or *Salmonella enterica* Paratyphi A were isolated from the blood of patients with culture-confirmed enteric fever.

Between Sept 18, 2011, and July 14, 2014, we screened 725 patients with suspected enteric fever for enrolment (figure 1). The data and safety monitoring board reviewed the outcome data after 100 patients, and then 200 patients, were randomised. At the 200-patient review, the board requested an additional review within 3 months of MICs against ciprofloxacin and gatifloxacin against all bacterial isolates. Data from 109 culture-confirmed patients (and 233 patients in total) were analysed at this additional review. A comparison of treatment failures between treatment groups did not cross the predefined Haybittle-Peto stopping boundary^23^ of p less than 0·001 (overall or in any subgroup), but the emergence of *S* Typhi strains with MICs against ciprofloxacin that were greater than 16 μg/mL and against gatifloxacin that were greater than 1 μg/mL, and a significant difference (p=0·0002 for susceptive *vs* resistant strains in the gatifloxacin group) in treatment response between patients with fluoroquinolone-resistant strains and those with susceptible strains, led the board to recommend stopping the trial on clinical grounds supported by data on the changing in-vitro susceptibility.

The trial was stopped on July 14, 2014. At this point, we had recruited and randomly assigned 246 eligible patients to treatment (including 116 patients with microbiologically confirmed disease; figure 1). Seven patients were excluded from the ITT population—four withdrew consent after randomisation but before receiving the first dose of study drug, and three had an alternative diagnosis. On stopping the trial, 120 patients had received gatifloxacin and 119 patients had received ceftriaxone, totalling 239 analysed in the modified ITT population. *S* Typhi or *S* Paratyphi A was isolated from the blood of 62 patients in the gatifloxacin group and 54 patients in the ceftriaxone group (figure 1). Analyses were not adjusted for early stopping of the trial.

The baseline characteristics of patients were balanced between the two treatment groups in the modified ITT population (table 1) except for a larger proportion of men in the gatifloxacin group. Similar numbers of patients in each group had received a non-exclusion antimicrobial in the 2 weeks before randomisation. However, culture-negative patients were more likely to have had enteric fever previously and to report coughing, and had lower serum transaminase concentrations, than patients with blood culture-confirmed *S* Typhi or *S* Paratyphi A (appendix). Moreover, patients with *S* Typhi were more likely to report anorexia, nausea, and diarrhoea, and had a lower haematocrit compared with the other two patient groups.

The MICs against ciprofloxacin and the study drugs were also balanced between the treatment groups (table 2). The first patient with a ciprofloxacin-resistant *S* Typhi culture (MIC >32 μg/mL) was enrolled on April 30, 2013. From that date, 118 additional patients were recruited, 55 of whom had positive blood cultures. Among these, 14 (25%) of 55 patients with *S* Typhi infections with high MICs against ciprofloxacin (12 >32 μg/mL, two 24 μg/mL) were assigned to a study drug; all 14 strains were also highly resistant to gatifloxacin (MIC ≥1·5 μg/mL).

**Figure 2:**
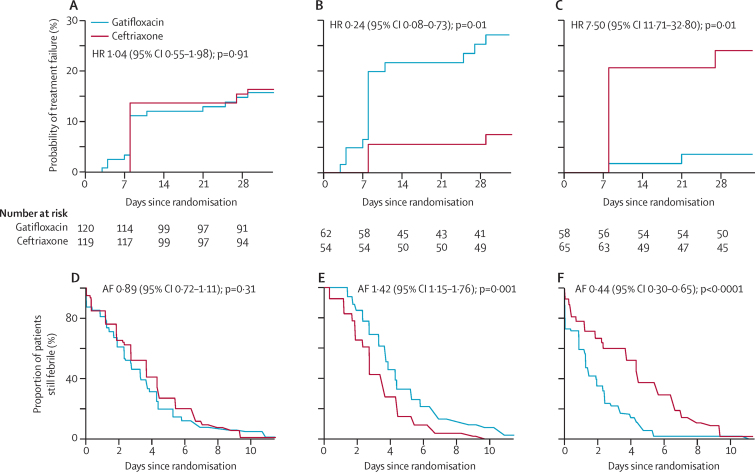
Time to treatment failure and fever clearance time Time to treatment failure shown in the (A) modified intention to treat, (B) culture-confirmed, and (C) culture-negative populations. Fever clearance times shown in the (D) modified intention to treat, (E) culture-confirmed, and (F) culture-negative populations. Fever clearance times were interval-censored; because numbers at risk are not well defined in this setting they are not shown for graphs D, E, or F. HR=hazard ratio. AF=acceleration factor.

Treatment failure in the modified ITT population was similar between treatment groups: 18 (15%) of 120 patients who received gatifloxacin had treatment failure, compared with 19 (16%) of 119 who received ceftriaxone (hazard ratio [HR] of time to failure 1·04 [95% CI 0·55–1·98]; p=0·91 [table 3]). Details for each event in the composite endpoint are in the appendix. However, there was significant heterogeneity in the primary outcome between the subpopulations of blood culture-confirmed and culture-negative patients (p_interaction_<0·0001; table 3, figure 2). In the culture-confirmed population, 16 (26%) of 62 patients given gatifloxacin had treatment failure, compared with four (7%) of 54 patients given ceftriaxone (HR 0·24 [95% CI 0·08–0·73, p=0·01; table 3, absolute risks of failure in appendix). For the four patients with treatment failure in the ceftriaxone group, MICs against ceftriaxone ranged from 0–0·2 μg/mL, and were similar to MICs in patients without treatment failure. Treatment failure was associated with the emergence of *S* Typhi exhibiting resistance against fluoroquinolones. None of the subgroup analyses for culture-positive patients showed significant treatment effect heterogeneity of the primary endpoint (table 3).

By contrast, in culture-negative patients, only two (3%) of 58 who received gatifloxacin failed treatment compared with 15 (23%) of 65 who received ceftriaxone (HR 7·50 [95% CI 1·71–32·80]; p=0·01 [table 3, absolute risks of failure in appendix]). The most common cause of treatment failure in culture-negative patients treated with ceftriaxone was fever for more than 7 days (12 [80%] of 15 patients) and nine [60%] of 15 received rescue treatment (appendix).

In the modified ITT population, fever clearance times did not differ between the two treatment groups (table 4, figure 2). Furthermore, the incidence of microbiological failure or syndromic relapse at any time until 6 months did not differ between the two treatment groups by day 28 or by 6 months (appendix).

We noted significant heterogeneity (p_interaction_<0·0001) of the treatment effect for fever clearance times in the blood culture-positive and blood culture-negative subgroups (table 4, figure 2). In culture-positive patients, median fever clearance times were longer in those treated with gatifloxacin than ceftriaxone (p=0·001) and outcomes with gatifloxacin were worse for patients with a raised MIC against ciprofloxacin and gatifloxacin (table 4). Occurrence of relapse did not differ between treatment groups in culture-positive patients (appendix). At 1-month follow-up, only two patients had positive stool cultures (one for *S* Typhi and one for *S* Paratyphi A), both in the gatifloxacin group. We noted no positive stool cultures at 3 months or 6 months in culture-positive patients (appendix). In culture-negative patients, fever clearance times were shorter in those treated with gatifloxacin than ceftriaxone (p<0·0001; table 4), but occurrences of relapse did not differ between treatment groups (appendix).

Over the course of the trial, 122 adverse events occurred in the gatifloxacin group and 120 in the ceftriaxone group (appendix); no serious adverse events were reported. The most common adverse events reported were vomiting (in 27 [23%] of 120 patients receiving gatifloxacin and 17 [14%] of 119 receiving ceftriaxone; p=0·13), and cough (which did significantly differ between groups: in 15 [12%] patients receiving gatifloxacin and 29 [24%] patients receiving ceftriaxone; p=0·02). No adverse events were attributed to any of the study treatments. The frequency of dysglycaemia and abnormal liver-function tests did not differ between the treatment groups (appendix), and none of the study participants died.

## Discussion

In patients with clinically suspected enteric fever, we showed that outcomes did not differ between patients receiving gatifloxacin and those receiving ceftriaxone. However, patients with blood culture-confirmed enteric fever fared less well when given gatifloxacin, as suggested by an increased likelihood of treatment failure and protracted fever clearance times. This finding was apparent only in the last year of recruitment into the trial as *S* Typhi strains with high-level resistance to ciprofloxacin and gatifloxacin (MICs >16 μg/mL and >1 μg/mL, respectively) emerged during the study, leading to the trial being stopped in July, 2014.

The data resulting from this trial contradict our hypothesis, because before the emergence of fluoroquinolone-resistant *S* Typhi in Nepal, we had shown in a series of clinical trials (done between 2004 and 2011) that gatifloxacin was both a safe and effective treatment for uncomplicated enteric fever.^14^, ^16^, ^17^ This antimicrobial has provided good clinical outcomes despite the continuing isolation of *S* Typhi and *S* Paratyphi A organisms showing reduced susceptibility against ciprofloxacin (MICs from 0·1 μg/mL–1 μg/mL).^7^, ^24^ Although the treatment failure results with gatifloxacin in our study are based on data from a few patients, these findings are highly consistent with the results of larger numbers for the secondary endpoint of fever clearance, which lend support to the credibility of our results.

Generally, we still regard gatifloxacin as a safe drug for use in this setting since we have no evidence for an increased risk of dysglycaemia or hepatitis. However, our new data suggest that the efficacy of gatifloxacin (and older-generation fluoroquinolones) for the treatment of enteric fever in Nepal is now compromised by the emergence of high-level fluoroquinolone-resistant *S* Typhi. As a result, we no longer advocate gatifloxacin as a treatment for enteric fever in Nepal.

Despite being a two-centre study in Kathmandu, our findings raise substantial questions regarding the use of fluoroquinolones in south Asia and other endemic regions for treating enteric fever. Genomic data has shown that strains with reduced susceptibility to fluoroquinolones are now globally dominant.^25^ The process of resistance development against the fluoroquinolones is a stepwise process; mutations are sequentially acquired in the target genes, thus determining higher MICs.^7^ Data obtained from in-vitro experiments suggest that strains with fluoroquinolone-resistance-associated mutations might actually have a selective advantage over wild-type strains.^26^ Fluoroquinolone resistance is clearly increasing, not only in Nepal, but also in neighbouring areas and other parts of the world where resources are low.^24^ Therefore in view of this combination of evidence, we predict a short window before the international emergence of *S* Typhi, and potentially *S* Paratyphi A, strains with high-level fluoroquinolone resistance, thus rendering this important group of antimicrobials globally ineffective for enteric fever. Conversely, the outcomes for ceftriaxone-treated, culture-positive patients were good, with short fever clearance times and few relapses. The optimum duration of ceftriaxone treatment is not clearly defined; in WHO guidelines, it is recommended for 10–14 days,^6^, ^19^ but our data lend support to a 7-day treatment course for patients with uncomplicated enteric fever in an endemic setting.

Alongside antimicrobial-resistant *S* Typhi, our clinical findings pose an additional clinical and public health challenge regarding ceftriaxone treatment. In patients with a negative blood-culture result, the absolute risk of treatment failure was 0·24 in the ceftriaxone group versus 0·04 in the gatifloxacin group. Furthermore, the median fever clearance times in this group were 3·03 days in the ceftriaxone group, versus 1·12 days in the gatifloxacin group. These contrasting outcomes for ceftriaxone in the two predefined patient populations were not predicted and previous similar data from other enteric fever trials are scarce. The reason for this shortage of data is because the results of patients enrolled in enteric fever trials who did not have a positive blood culture were, until recently, not reported. From a small ceftriaxone study^11^ done in Vietnamese patients with enteric fever, the investigators reported that two of the six culture-negative patients given ceftriaxone failed treatment, but responded to rescue treatment with ofloxacin. Only four randomised trials for enteric fever^14^, ^15^, ^16^, ^17^ did an intention-to-treat analysis and incorporated an analysis for the blood culture-negative patients. In these trials, culture-negative patients given gatifloxacin, ofloxacin, or azithromycin achieved similar (or better) outcomes than those reported in blood culture-confirmed patients with enteric fever.

Better clinical outcomes in patients with syndromic enteric fever but with a negative blood-culture result might be attributed to the low sensitivity of blood-culture tests (estimated to be 50–60%)^5^ and the possibility of fewer bacteria in the bloodstream, corresponding with less severe disease. We do not know how many culture-negative patients actually had enteric fever in our study; some might have had alternative bacterial infections. Our previous study^27^ examined archived blood samples from 765 adults presenting at Patan Hospital, Nepal, with undifferentiated febrile illness in 2001. 50 (7%) patients had *Rickettsia typhi* (murine typhus) DNA detected by PCR amplification. Furthermore, we investigated the infectious cause of culture-negative patients enrolled into one of our other enteric fever trials in Nepal and noted serological evidence of murine typhus in 21 (22%) of 96 blood culture-negative patients, with 12 (57%) of 21 testing positive for *R typhi* with PCR amplification.^28^ Thus, we surmise that a substantial proportion of culture-negative patients with suspected enteric fever in Nepal might actually have other bacterial infections, included those caused by the rickettsiaceae. Ceftriaxone is not regarded as an effective treatment for murine typhus and other rickettsial illnesses, whereas fluoroquinolones do seem to have clinical activity against these pathogens.^28^ However, no rapid diagnostic tests are available that can accurately differentiate between rickettsial infections, enteric fever, or indeed any bacteraemia, and inform patient management in a timely manner. Without such a test, we suggest that a more pragmatic approach would be to combine ceftriaxone with doxycycline for patients without a positive blood culture who do not respond to ceftriaxone monotherapy.

In conclusion, the results of our trial underline two substantial problems for patients with enteric fever. First, the continued development of antimicrobial resistance in the pathogens causing the disease; second, the absence of point-of-care diagnostic tests for febrile diseases in low-income settings.^29^ We have shown that high-level fluoroquinolone-resistant *S* Typhi is now likely to be endemic in Nepal and suggest that fluoroquinolones—even the fourth-generation fluoroquinolone gatifloxacin—cannot be recommended as empirical therapy for enteric fever in this setting. Azithromycin and ceftriaxone are alternative options, although sporadic cases of resistance have been reported, and data comparing in-vitro azithromycin susceptibility against clinical outcomes are poor.^30^, ^31^ Additionally, under our study conditions, ceftriaxone was suboptimum in a large proportion of culture-negative patients with suspected enteric fever, which further emphasises the need for diagnostic tests for enteric fever and other common febrile diseases. Since antimicrobials, specifically fluoroquinolones, are one of the only routinely used control measures for enteric fever, effective surveillance programmes, the assessment of novel diagnostics, new treatment options, and the use of existing vaccines and development of next-generation vaccines are now a greater priority than ever.

## Figures and Tables

**Table 1:** Baseline characteristics of the modified intention-to-treat population

		**Gatifloxacin (N=120)**		**Ceftriaxone (N=119)**	
		N	n (%) or median (IQR)	N	n (%) or median (IQR)
Age (years)		120	19·0 (15·0−23·0)	119	20·0 (14·0−23·5)
Sex					
	Male	120	99 (83%)	119	81 (68%)
	Female	120	21 (18%)	119	38 (32%)
Temperature (°C)		116	38·8 (38·3−39·4)	116	38·8 (38·3−39·4)
Days of illness before enrolment		120	5·0 (4·0−6·0)	119	5·0 (4·0−7·0)
Treatment with antibiotics in past 2 weeks		120	21 (18%)	119	17 (14%)
Previous history of typhoid		120	18 (15%)	118	19 (16%)
Family history of typhoid		120	18 (15%)	119	17 (14%)
Typhoid vaccination		119	6 (5%)	119	5 (4%)
Fever		120	120 (100%)	118	118 (100%)
Cough		115	38 (33%)	113	42 (37%)
Constipation		117	9 (8%)	116	16 (14%)
Headache		119	99 (83%)	116	108 (93%)
Diarrhoea		117	25 (21%)	116	28 (24%)
Vomiting		116	32 (28%)	116	30 (26%)
Abdominal pain		114	31 (27%)	115	27 (23%)
Anorexia		118	88 (75%)	116	80 (69%)
Nausea		116	60 (52%)	114	55 (48%)
Splenomegaly		117	0	114	2 (2%)
Hepatomegaly		117	0	114	0
Random blood glucose (mmol/L)		117	5·38 (4·77−6·11)	117	5·38 (4·94−5·88)
Creatinine (μmol/L)		116	70·72 (61·88−79·56)	114	70·72 (61·88−79·56)
Total bilirubin (μmol/L)		117	13·68 (10·26−17·10)	117	11·97 (10·26−15·39)
Leucocyte cell count (×10^9^/L)		120	6·9 (4·8−7·2)	119	5·8 (4·7−7·3)
Haematocrit (%)		119	39·6 (37·0−43·0)	116	38·7 (35·8−44·0)
Platelet cell count (×10^9^/L)		120	170·0 (150·0−210·0)	119	167·0 (145·5−203·0)
AST (U/L)		117	46·0 (32·0−66·0)	116	51·5 (38·8−80·0)
ALT (U/L)		117	46·0 (30·0−63·0)	117	45·0 (33·0−63·0)
Culture positive					
	*Salmonella* Paratyphi A isolated	120	19 (16%)	119	16 (13%)
	*Salmonella* Typhi isolated	120	43 (36%)	119	38 (32%)
No growth or culture negative		120	58 (48%)	119	65 (55%)

N refers to the number of patients with non-missing data in each group. AST=serum aspartate aminotransferase. ALT=serum alanine aminotransferase.

**Table 2:** Minimum inhibitory concentration of organism in the culture-confirmed population at enrolment

			**All patients**	**Gatifloxacin**	**Ceftriaxone**
*Salmonella* Typhi			N=81	N=43	N=38
	MIC against ciprofloxacin (μg/mL)		n=78	n=41	n=37
		MIC 50	0·38	0·38	0·38
		MIC 90	>32·00	>32·00	13·40
		Range*	0·008–>32·00	0·008–>32·00	0·016–>32·00
	MIC against gatifloxacin (μg/mL)		n=78	n=41	n=37
		MIC 50	0·125	0·125	0·125
		MIC 90	2·000	2·000	1·250
		Range*	0·006−3·000	0·006−3·000	0·006−3·000
	MIC against ceftriaxone (μg/mL)		n=78	n=41	n=37
		MIC 50	0·094	0·094	0·125
		MIC 90	0·190	0·190	0·190
		Range*	0·032−0·640	0·032−0·250	0·047−0·640
*Salmonella* Paratyphi A			N=35	N=19	N=16
	MIC against ciprofloxacin (μg/mL)		n=34	n=18	n=16
		MIC 50	0·500	0·625	0·500
		MIC 90	0·925	1·000	0·750
		Range*	0·380−1·000	0·380−1·000	0·380−1·000
	MIC against gatifloxacin (μg/mL)		n=34	n=18	n=16
		MIC 50	0·500	0·500	0·500
		MIC 90	0·750	0·575	0·750
		Range*	0·380−0·750	0·380−0·750	0·380−0·750
	MIC against ceftriaxone (μg/mL)		n=34	n=18	n=16
		MIC 50	0·125	0·125	0·125
		MIC 90	0·190	0·145	0·220
		Range*	0·064−0·500	0·094−0·190	0·064−0·500

n refers to the number of patients with non-missing data in each group. MIC=minimum inhibitory concentration. MIC 50=minimum inhibitory concentration at the 50th percentile. MIC 90=minimum inhibitory concentration at the 90th percentile.

* Range from the minimum to the maximum noted MIC.

**Table 3:** Treatment failure (primary endpoint) overall and in predefined subgroups

		**Gatifloxacin (events/n [%])**	**Ceftriaxone (events/n [%])**	**Hazard ratio of time to failure (95% CI); p value**	**Heterogeneity test (p_interaction_ value)**
All patients (modified intention-to-treat population)		18/120 (15%)	19/119 (16%)	1·04 (0·55−1·98); p=0·91	
Culture-negative or culture-positive populations					<0·0001
	Culture negative	2/58 (3%)	15/65 (23%)	7·50 (1·71−32·80); p=0·01	
	Culture positive	16/62 (26%)	4/54 (7%)	0·24 (0·08−0·73); p=0·01	
Pathogen (culture-confirmed population)					0·25
	*Salmonella* Paratyphi A	1/19 (5%)	1/16 (6%)	1·13 (0·07−18·02); p=0·93	
	*Salmonella* Typhi	15/43 (35%)	3/38 (8%)	0·18 (0·05−0·62); p=0·01	
Age (modified intention-to-treat population)					0·25
	<16 years	6/32 (19%)	4/36 (11%)	0·57 (0·16−2·00); p=0·38	
	≥16 years	12/88 (14%)	15/83 (18%)	1·31 (0·61−2·80); p=0·48	
Age (culture-confirmed population)					0·76
	<16 years	6/21 (29%)	1/16 (6%)	0·19 (0·02−1·62); p=0·13	
	≥16 years	10/41 (24%)	3/38 (8%)	0·27 (0·07−0·98); p=0·047	
Sex (modified intention-to-treat population)					0·52
	Female	3/21 (14%)	4/38 (11%)	0·69 (0·15−3·07); p=0·62	
	Male	15/99 (15%)	15/81 (19%)	1·21 (0·59−2·47); p=0·61	
Sex (culture-confirmed population)					0·08
	Female	3/11 (27%)	0/17	0 (0–∞); p=1·00	
	Male	13/51 (25%)	4/37 (11%)	0·37 (0·12−1·15); p=0·09	
Recruitment date (modified intention-to-treat population)					0·15
	Before April 1, 2013	7/62 (11%)	11/59 (19%)	1·69 (0·66−4·36); p=0·28	
	April 1, 2013, or later	11/58 (19%)	8/60 (13%)	0·65 (0·26−1·61); p=0·35	
Recruitment date (culture-confirmed population)					0·70
	Before April 1, 2013	6/33 (18%)	1/28 (4%)	0·18 (0·02−1·46); p=0·11	
	April 1, 2013, or later	10/29 (34%)	3/26 (12%)	0·28 (0·08−1·00); p=0·05	
MIC against ciprofloxacin (culture-confirmed population)					0·15
	<0·12 μg/mL	0/4	1/3 (33%)	∞ (0–∞); p=1·00	
	0·12−2·00 μg/mL	8/45 (18%)	2/46 (4%)	0·22 (0·05−1·05); p=0·06	
	>2·00 μg/mL*	8/10 (80%)	1/4 (25%)	0·17 (0·02−1·38); p=0·10	
MIC against gatifloxacin (culture-confirmed population)					0·58
	≤1 μg/mL	8/49 (16%)	3/49 (6%)	0·34 (0·09−1·28); p=0·11	
	>1 μg/mL	8/10 (80%)	1/4 (25%)	0·17 (0·02−1·38); p=0·10	

MIC=minimum inhibitory concentration.

* Among the 14 strains, two had a ciprofloxacin MIC of 24 μg/mL and 12 had a ciprofloxacin MIC>32 μg/mL.

**Table 4:** Fever clearance time (secondary endpoint) overall and in predefined subgroups

		**Gatifloxacin**		**Ceftriaxone**		**Acceleration factor (95% CI), p value**	**Heterogeneity test (p_interaction_ value)**
		n	Median (IQR) days	n	Median (IQR) days		
All patients (modified intention-to-treat population)		120	2·43 (1·09−4·56)	119	2·93 (1·44−5·12)	0·89 (0·72−1·11); p=0·31	
Culture-negative or culture-positive population							<0·0001
	Culture negative	58	1·12 (0·39−2·58)	65	3·03 (1·31−5·85)	0·44 (0·30−0·65); p<0·0001	
	Culture positive	62	4·21 (2·63−6·10)	54	2·78 (1·62−4·26)	1·42 (1·15−1·76); p=0·001	
Pathogen (culture-confirmed population)							0·57
	*Salmonella* Paratyphi A	19	3·68 (2·50−4·98)	16	2·24 (1·18−3·69)	1·31 (0·88−1·94); p=0·19	
	*Salmonella* Typhi	43	4·51 (2·79−6·58)	38	3·03 (1·86−4·46)	1·47 (1·15−1·88); p=0·002	
Age (modified intention-to-treat population)							0·29
	<16 years	32	3·02 (1·70−4·75)	36	2·23 (0·93−4·46)	1·08 (0·72−1·60); p=0·72	
	≥16 years	88	2·22 (0·92−4·45)	83	3·25 (1·70−5·41)	0·83 (0·64−1·08); p=0·16	
Age (culture-confirmed population)							0·46
	<16 years	21	3·82 (2·45−5·43)	16	3·04 (1·94−4·32)	1·26 (0·90−1·76); p=0·18	
	≥16 years	41	4·43 (2·77−6·43)	38	2·66 (1·47−4·22)	1·51 (1·15−1·97); p=0·003	
Sex (modified intention-to-treat population)							0·99
	Female	21	2·41 (1·13−4·39)	38	2·78 (1·34−4·94)	0·89 (0·56−1·41); p=0·61	
	Male	99	2·44 (1·09−4·59)	81	2·99 (1·48−5·20)	0·89 (0·68−1·14); p=0·35	
Sex (culture-confirmed population)							0·68
	Female	11	4·27 (3·12−5·47)	17	3·06 (1·93−4·41)	1·25 (0·86−1·83); p=0·25	
	Male	51	4·18 (2·56−6·16)	37	2·66 (1·50−4·17)	1·46 (1·13−1·89); p=0·004	
Recruitment date (modified intention-to-treat population)							0·09
	Before April 1, 2013	62	2·30 (1·10−4·09)	59	2·79 (1·16−5·55)	0·74 (0·53−1·03); p=0·08	
	April 1, 2013, or later	58	2·60 (1·12−5·04)	60	3·05 (1·76−4·69)	1·09 (0·82−1·45); p=0·56	
Recruitment date (culture-confirmed population)							0·12
	Before April 1, 2013	33	3·88 (2·63−5·26)	28	2·54 (1·31−4·27)	1·21 (0·90−1·63); p=0·22	
	April 1, 2013, or later	29	4·68 (2·82−6·97)	26	3·00 (1·96−4·20)	1·68 (1·26−2·23); p=0·0004	
MIC against ciprofloxacin (culture-confirmed population)							0·02
	<0·12 μg/mL	4	2·55 (1·82−3·32)	3	4·98 (4·09−5·82)	0·58 (0·35−0·94); p=0·028	
	0·12−2·00 μg/mL	45	3·88 (2·67−5·21)	46	2·63 (1·49−4·12)	1·24 (0·99−1·56); p=0·06	
	>2·00 μg/mL*	10	8·20 (5·99−10·50)	4	3·66 (2·84−4·46)	2·36 (1·58−3·51); p=<0·0001	
MIC against gatifloxacin (culture-confirmed population)							0·049
	≤1·00 μg/mL	49	3·76 (2·56−5·08)	49	2·75 (1·58−4·27)	1·17 (0·94−1·45); p=0·15	
	>1·00 μg/mL	10	8·20 (5·99−10·50)	4	3·66 (2·84−4·46)	2·36 (1·58−3·51); p<0·0001	

Percentages not added to this table because the denominators for populations change and are not clearly specified. MIC=minimum inhibitory concentration.

* Among the 14 strains, two had a ciprofloxacin MIC of 24 μg/mL and 12 had an MIC >32 μg/mL.
