# Supplementary material for: Gatifloxacin versus ceftriaxone for uncomplicated enteric fever in Nepal: an open-label, two-centre, randomised controlled trial
Source: Lancet Infect Dis. 2016 May;16(5):535–45. doi: 10.1016/S1473-3099(15)00530-7 (PMC4835582; doi:10.1016/S1473-3099(15)00530-7)
Supplement: Supplementary appendix [file mmc1.pdf]

THE LANCET Infectious Diseases

Supplementary webappendix

This webappendix formed part of the original submission and has been peer reviewed. We post it as supplied by the authors.

Supplement to: Arjyal A, Basnyat B, Nhan HT, et al. Gatifloxacin versus ceftriaxone for uncomplicated enteric fever in Nepal: an open-label, two-centre, randomised controlled trial. *Lancet Infect Dis* 2016; published online Jan 19. http://dx.doi.org/10.1016/S1473-3099(15)00530-7.

Supplementary table 1. Baseline characteristics by culture result

**Baseline characteristics**	**n**	**Culture-negative (N=123)**	**n**	***Salmonella* Paratyphi A (N=35)**	**n**	***Salmonella* Typhi (N=81)**	**Comparison (p-value) ***
Age (years) – median (IQR)	123	20·0(15·5,26·0)	35	19·0(12·0,24·0)	81	18·0(13·0,22·0)	0·07
Sex (male)	123	92 (74·8%)	35	29 (82·9%)	81	59 (72·8%)	0·50
Temperature (°C) - median (IQR)	119	38·8 (38·3,39·4)	35	38·8 (38·3,39·0)	78	39·0 (38·3,39·4)	0·46
Days of illness before enrolment - median (IQR)	123	5·0(4·0,6·0)	35	5·0(4·0,6·5)	81	5·0(4·0,7·0)	0·24
Antibiotics treatment in last two weeks	123	20 (16·3%)	35	6 (17·1%)	81	12 (14·8%)	0·94
Previous history of typhoid	123	27 (22·0%)	34	4 (11·8%)	81	6 (7·48%)	0·02
Family history of typhoid	123	21 (17·1%)	35	6 (17·1%)	81	8 (9·9%)	0·34
Typhoid vaccination	122	7 (5·7%)	35	3 (8·6%)	81	1 (1·2%)	0·12
Fever	123	123 (100%)	35	35 (100%)	80	80 (100%)	1·00
Cough	117	51 (43·6%)	34	7 (20·6%)	77	22 (28·6%)	0·02
Constipation	120	11 (9·2%)	35	8 (22·9%)	78	6 (7·7%)	0·06
Headache	120	102 (85·0%)	35	31 (88·6%)	80	74 (92·5%)	0·29
Diarrhoea	120	17 (14·2%)	35	5 (14·3%)	78	31 (39·7%)	0·0001
Vomiting	119	31 (26·1%)	35	9 (25·7%)	78	22 (28·2%)	0·94
Abdominal pain	117	26 (22·2%)	35	9 (25·7%)	77	23 (29·9%)	0·48
Anorexia	120	78 (65·0%)	35	24 (68·6%)	79	66 (83·5%)	0·01
Nausea	120	54 (45·0%)	34	13 (38·2%)	76	48 (63·2%)	0·02
Splenomegaly	120	1 (0·8%)	32	1 (3·1%)	79	0 (0%)	0·37
Hepatomegaly	120	0(0%)	32	0(0%)	79	0(0%)	1·00
Random blood glucose (mmol/L) - median (IQR)	120	5.47(4.83, 6.11)	35	5.33(4.77, 5.63)	79	5.38(4.94, 6.02)	0.61
Creatinine (μmol/L) - median (IQR)	118	70.72(61.88, 79.56)	35	70.72(53.04, 79.56)	77	70.72(61.88, 79.56)	0.78
Total Bilirubin (μmol/L)- median (IQR)	119	13.68(10.26, 17.10)	35	11.97(10.26, 17.10)	80	11.97(8.55, 15.39)	0.36
Leucocyte count ($\times 10^9$/L) - median (IQR)	123	5·9 (4·7,7·5)	35	5·7(4·7,6·7)	81	5·9 (4·8,6·8)	0·36
Haematocrit (%) - median (IQR)	122	40·0 (36·9,44·0)	35	40·8(37·6,44·0)	78	38·0 (35·2,41·4)	0·02
Platelet count ($\times 10^9$/L) - median (IQR)	123	172·0(150·0,214·5)	35	171·0(150·5,203·0)	81	162·0(146·0,204·0)	0·40

AST (U/L) - median (IQR)	120	41·0(30·0,58·2)	35	56·0(41·5,79·0)	78	62·0(44·0,80·0)	<0·0001
ALT (U/L) - median (IQR)	120	38·0(27·5,55·2)	35	47·0(35·5,61·5)	79	51·0(40·5,67·0)	0·0003

*Comparison between all three groups was done using Fisher's exact test for categorical variables and the Kruskal-Wallis test for continuous variables.

AST=serum aspartate aminotransferase. ALT=serum alanine aminotransferase. IQR=inter-quartile range.

n refers to the number of subjects with non-missing data.

Supplementary table 2. Summary of primary and secondary endpoints for the intention to treat population

	**Gatifloxacin (N=120)**	**Ceftriaxone (N=119)**	**Effect size(95%CI); p-value**
Treatment failure *	18	19	HR=1·04 (0·55,1·98); p=0·91
- Fever over 7days	9	14	
- Rescue treatment	10	10	
- Microbiological failures	2	1	
- Enteric fever related complications $	1	0	
- Relapse until day 28	5	3	
Risk of failure until day 28 †	0·15 (95%CI:0·09,0·22)	0·16 (95%CI:0·09,0·23)	RD=0·01(-0·09,0·10);p=0·89
Median (IQR) time to fever clearance (days) #	2·43(1·09,4·56)	2·93(1·44,5·12)	AF=0·89(0·72,1·11); p=0·31
Relapse until day 28	5	3	HR=0·56 (0·13,2·36); p=0·43
- Confirmed relapse until day 28	4	3	
- Syndromic relapse until day 28	1	0	
Relapse at any time until 6 months €	10	7	HR=0·67(0·25,1·75); p=0·41
- Confirmed relapse at any time until 6 months	8	4	
- Syndromic relapse at any time until 6 months	2	3	

HR=hazard ratio of time to event (based on Cox regression). RD=absolute risk difference (based on Kaplan-Meier estimates). AF= acceleration factor (based on a Weibull accelerated failure time model for interval-censored fever clearance time).

*Patients can have more than one type of treatment failure. $ This patient was admitted to hospital.

†Kaplan-Meier estimates. #Estimated from Weibull accelerated failure time model. € All relapses occurred within 2 months of enrolment except for a single syndromic relapse on ceftriaxone which occurred on day 142.

Supplementary table 3. Summary of primary and secondary endpoints for the culture-confirmed population

	**Gatifloxacin (N=62)**	**Ceftriaxone (N=54)**	**Effect size(95%CI); p-value**
Treatment failure *	16	4	HR=0·24(0·08,0·73); p=0·01
- Fever over 7days	8	2	
- Rescue treatment	9	1	
- Microbiological failures	2	1	
- Enteric fever related complications $	1	0	
- Relapse until day 28	4	1	
Risk of failure until day 28 †	0·27 (95%CI:0·15,0·38)	0·07 (95%CI:0·00,0·14)	RD= -0·20(-0·33,-0·06);p=0·004
Median (IQR) time to fever clearance (days) #	4·21(2·63,6·1)	2·78(1·62,4·26)	AF=1·42(1·15,1·76); p=0·001
Relapse until day 28	4	1	HR=0·24(0·027,2·16); p=0·20
- Confirmed relapse until day 28	4	1	
- Syndromic relapse until day 28	0	0	
Relapse at any time until 6 months €	6	4	HR=0·64(0·18,2·26);p=0·48
- Confirmed relapse at any time until 6 months	5	1	
- Syndromic relapse at any time until 6 months	1	3	
Faecal carriage in stool £			
- Month 1	2/35	0/38	-
- Month 3	0/34	0/26	-
- Month 6	0/24	0/22	-

HR= hazard ratio of time to event (based on Cox regression). RD=absolute risk difference (based on Kaplan-Meier estimates). AF= acceleration factor (based on a Weibull accelerated failure time model for interval-censored fever clearance time).

*Patients can have more than one type of treatment failure. $ This patient was admitted to hospital.

†Kaplan-Meier estimates. # Estimated from Weibull accelerated failure time model. € All relapses occurred within 2 months of enrolment except for a single syndromic relapse on ceftriaxone which occurred on day 142.

£ Denominators correspond to the number of patients with a stool sample at each time point. Faecal carriage was only descriptively analyzed. At month 1, one of the samples was positive for S.Paratyphi A and one for S.Typhi.

Supplementary table 4. Summary of primary and secondary endpoints for the culture-negative population

	**Gatifloxacin (N=58)**	**Ceftriaxone (N=65)**	**Effect size(95%CI);p-value**
Treatment failure *	2	15	HR=7·50(1·71,32·80); p=0·01
- Fever over 7days	1	12	
- Rescue treatment	1	9	
- Microbiological failures	0	0	
- Enteric fever related complications	0	0	
- Relapse until day 28	1	2	
Risk of failure until day 28 †	0·04 (95%CI:0·00,0·08)	0·24 (95%CI:0·13,0·34)	RD=0·20(0·09,0·32);p=0·001
Median (IQR) time to fever clearance (days) #	1·12(0·39,2·58)	3·03(1·31,5·85)	AF=0·44(0·30,0·65); p<0·0001
Relapse until day 28	1	2	HR=1·81(0·16,20·01); p=0·63
- Confirmed relapse until day 28	0	2	
- Syndromic relapse until day 28	1	0	
Relapse at any time until 6 months €	4	3	HR=0·71(0·16,3·18); p=0·66
- Confirmed relapse at any time until 6 months	3	3	
- Syndromic relapse at any time until 6 months	1	0	

HR= hazard ratio of time to event (based on Cox regression). RD=absolute risk difference (based on Kaplan-Meier estimates). AF= acceleration factor (based on a Weibull accelerated failure time model for interval-censored fever clearance time).

*Patients can have more than one type of treatment failure.

†Kaplan-Meier estimates. # Estimated from Weibull accelerated failure time model. € All relapses occurred within 2 months of enrolment.

Supplementary tables 5. Laboratory abnormalities after enrolment: random blood glucose (intention to treat population)

Worst random blood glucose grade	n	Gatifloxacin (N=120)	N	Ceftriaxone (N=119)	Comparison (p-value)*
Worst hyperglycemia grade	120		118		0·98
- Grade 0		10 (8·3%)		11 (9·3%)	
- Grade 1		92 (76·7%)		87 (73·7%)	
- Grade 2		17 (14·2%)		20 (16·9%)	
- Grade 3		1 (0·8%)		0 (0·0%)	
Worst hypoglycemia grade	120		118		0·65
- Grade 0		105 (87·5%)		107 (90·7%)	
- Grade 1		13 (10·8%)		8 (6·8%)	
- Grade 2		2 (1·7%)		3 (2·5%)	

*Based on Cochran-Armitage trend test.

n refers to the number of subjects with at least one non-missing laboratory value after enrolment in each group.

Hyperglycemia: Grade 0 (≤115 mg/dL, no hyperglycemia), grade 1 (116-160 mg/dL), grade 2 (161-250 mg/dL), grade 3 (251-500 mg/dL), grade 4 (>500 mg/dL).

Hypoglycemia: Grade 0 (≥65 mg/dL, no hypoglycemia), grade 1 (55-64 mg/dL), grade 2 (40-54 mg/dL), grade 3 (30-39 mg/dL), grade 4 (< 30 mg/dL).

Supplementary tables 6. Laboratory abnormalities after enrolment: liver function (intention to treat population)

	n	Gatifloxacin (N=120)	n	Ceftriaxone (N=119)	Comparison (p-value)*
Worst ALT grade	113		109		0·18
- Grade 0		76 (67·3%)		67 (61·5%)	
- Grade 1		26 (23·0%)		26 (23·9%)	
- Grade 2		9 (8·0%)		10 (9·2%)	
- Grade 3		2 (1·8%)		6 (5·5%)	
Worst AST grade	112		109		0·11
- Grade 0		68 (60·7%)		53 (48·6%)	
- Grade 1		28 (25·0%)		35 (32·1%)	
- Grade 2		12 (10·7%)		16 (14·7%)	
- Grade 3		4 (3·6%)		5 (4·6%)	
Worst total bilirubin grade	107		106		0·09
- Grade 0		102 (95·3%)		105 (99·1%)	
- Grade 1		4 (3·7%)		1 (0·9%)	
- Grade 2		1 (0·9%)		0 (0·0%)	

*Based on Cochran-Armitage trend test.

n refers to the number of subjects with at least one non-missing laboratory value after enrolment in each group.

ALT=serum alanine aminotransferase: Upper limit of normal range (ULN): 69 U/L, grade 0 (no elevation, <1·25 UL), grade 1 (1·25 – 2·5 x ULN), grade 2 (2·6 – 5·0 x ULN), grade 3 (5·1 – 10·0 x ULN), grade 4 (> 10·0 x ULN).

AST=serum aspartate aminotransferase: ULN: 46 U/L, grade 0 (no elevation, <1·25 UL), grade 1 (1·25 – 2·5 x ULN), grade 2 (2·6 – 5·0 x ULN), grade 3 (5·1 – 10·0 x ULN), grade 4 (> 10·0 x ULN).

Bilirubin: ULN=1·3mg/dl, grade 0 (no elevation, <1·1 ULN), grade 1 (1·1 – 1·5 x ULN), grade 2 (1·6 – 2·5 x ULN), grade 3 (2·6 – 5·0 x ULN), grade 4 (> 5·0 x ULN).

table 7: Adverse events by treatment group (intention to treat population).

	**Gatifloxacin (n=120)**	**Ceftriaxone (n=119)**	**Comparison (p value)***
Patients with at least one adverse event	74 (61·7%)	74 (62·2%)	1·00
Total number of adverse events	122	120	-
Number of patients with specific events:			
- Anorexia	6 (5·0%)	2 (1·7%)	0·28
- Body pain	3 (2·5%)	3 (2·5%)	1·00
- Burning micturition	0 (0·0%)	1 (0·8%)	0·50
- Chest pain	1 (0·8%)	2 (1·7%)	0·62
- Chills/rigors	2 (1·7%)	5 (4·2%)	0·28
- Common cold	0 (0·0%)	1 (0·8%)	0·50
- Constipation	4 (3·3%)	1 (0·8%)	0·37
- Cough	15 (12·5%)	29 (24·4%)	0·02
- Diarrhoea	5 (4·2%)	12 (10·1%)	0·08
- Dizziness	11 (9·2%)	9 (7·6%)	0·82
- Eye pain	0 (0·0%)	1 (0·8%)	0·50
- Eyelid swelling	0 (0·0%)	1 (0·8%)	0·50
- Fever	1 (0·8%)	0 (0·0%)	1·00
- Headache	6 (5·0%)	1 (0·8%)	0·12
- Indigestion	0 (0·0%)	1 (0·8%)	0·50
- Insomnia	0 (0·0%)	1 (0·8%)	0·50
- Muscle pain	0 (0·0%)	1 (0·8%)	0·50
- Nasal bleeding	1 (0·8%)	0 (0·0%)	1·00
- Nausea	15 (12·5%)	9 (7·6%)	0·28
- Oral ulcer	0 (0·0%)	1 (0·8%)	0·50
- Pain abdomen	10 (8·3%)	18 (15·1%)	0·11
- Pain at injection site	0 (0·0%)	1 (0·8%)	0·50
- Rashes on body	1 (0·8%)	0 (0·0%)	1·00
- Sweating	1 (0·8%)	0 (0·0%)	1·00
- Throat pain	5 (4·2%)	1 (0·8%)	0·21
- Vertigo	5 (4·2%)	1 (0·8%)	0·21
- Vomiting	27 (22·5%)	17 (14·3%)	0·13

All reported adverse events were non-severe (i.e. grade 1 or grade 2).

*Based on Fisher's exact test.
